# Administrative Ethics Conflict and Governance of Grassroots Government Staff Under the Human Relationship Society

**DOI:** 10.3389/fpsyg.2022.842057

**Published:** 2022-05-09

**Authors:** Yue Yin, Taotao Li, Fan Yang

**Affiliations:** ^1^School of Public Administration and Law, Northeast Agricultural University, Harbin, China; ^2^Human Resources Office, Guangxi Open University, Nanning, China; ^3^School of Marxism, Mudanjiang Medical University, Mudanjiang, China

**Keywords:** administrative ethics conflicts, human relations, grassroots, civil servants, public administration

## Abstract

The conflict of administrative morality among civil servants at the grassroots level arises from the background of China’s long-standing traditional culture, and the current administrative system cannot keep up with the pace of economic development. In the process of grassroots management, due to the lag in the construction of administrative morality, the traditional official standard thinking, the imperfection of the current system, and the restriction of human nature, it is easy to cause the administrative moral conflict of the grassroots civil servants in practice. This paper takes the interpersonal society as the research background, analyzes the influence of the interpersonal society on the environment, and studies the administrative ethics conflicts and governance issues of basic civil servants from the interpersonal background. In addition, this paper conducts a more detailed field investigation based on fuzzy cluster analysis, analyzes the manifestations and causes of the administrative moral conflict of grassroots civil servants in the context of human society from multiple perspectives and levels, and then proposes countermeasures to solve the administrative moral conflict. The administrative moral conflict and governance of grass-roots civil servants under the background of interpersonal relationship is of great positive significance for solving the administrative moral conflict and improving the service awareness and service level of grass-roots civil servants.

## Introduction

As ethical individuals, the civil servants of exercising public power are actually a process of value selection, balance, and distribution according to ethical norms. Undoubtedly, civil servants are bound to affect by many ethical relationships while performing their duties. Due to etiquette and favors, some civil servants would not refuse to ask for lobbying, banquets for entertainment, and donations of property. However, it is often these interpersonal relationships, which breed corruption, such as abuse of power, the power for personal gain, and cronyism. In this way, how to reconcile the conflict between morality and power, and the conflict between human feelings and legal principles has become a problem that civil servants need to face (Fan et al., 2017). According to statistics in 2016, there were more than 71 million civil servants in China, of which grassroots civil servants accounted for about 60% of the total civil servants (Trondal et al., 2020). As an important executor of the government’s public policies, basic-level civil servants play an irreplaceable role in promoting economic development and promoting social stability. To a certain extent, the administrative level of grassroots civil servants directly affects the operational efficiency of government agencies and the public image.

At this stage, China is still a “human relationship society,” but it is different from the traditional “human relationship society” in the past. The traditional “human relationship society” is based on blood and geopolitical civil contacts. The research on administrative ethics in western countries originated in the 2010s and has a long history (Vargas et al., 2021). However, the research on the administrative ethics of grassroots civil servants is less involved, mostly from the perspective of the roles and responsibilities of administrative personnel. The administrative ethics conflict arises because the diversification of individual roles causes the conflict of responsibilities and obligations (Cui and Yan, 2020). The administrative ethical dilemma is mainly about choosing value and realizing public interests. The embryonic stage of the study of administrative ethics compared with foreign studies, many theoretical practices were immature, and there were relatively few studies on the administrative ethics conflicts of civil servants at the grassroots level in China. In terms of the connotation of administrative ethics conflict, when public administrators are dealing with a matter, they are aware of the incompatible requirements of two or more different ethical standards. Public administrators cannot comply with these requirements at the same time (Sydaxay and Wu, 2020). To make a choice, you will be in a difficult situation. The so-called ethical conflict refers to a dilemma when ethical subjects make ethical evaluation and ethical choices, which means the ability of ethical subjects to choose. When the public administrative body chooses one of the ethical norms when choosing behavior, it will violate the special state of the other ethical norm. Regarding the necessity of resolving administrative ethical conflicts, if civil servants fail to deal with administrative ethical conflicts correctly, they will be troubled by themselves (Malhotra et al., 2021). In serious cases, they will have an adverse impact on their mental health and personality, and even ethical behaviors. The manifestation and background of administrative ethical conflicts can provide guidance for effectively solving administrative ethical conflicts. In terms of the causes of administrative ethical conflicts, the essence of administrative ethics issues is not to stop at a nice slogan, but how to deal with administrative ethical conflicts and how to get out of the dilemma of administrative subjects’ ethical choices.

The problem of administrative ethics conflicts among grass-roots civil servants in the context of human relations and society arises under the background of China’s long traditional culture and the current administrative system cannot keep up with the pace of economic development (Wang et al., 2020). This article proposes fuzzy cluster analysis for contemporary social conflicts and contradictions. This article attempts to establish the “rule of law” construction based on the logic sequence of emotion, reason, and law of traditional social governance and the administrative ethics conflicts of grassroots civil servants under the social background of human relationship. As well as governance countermeasures, this paper combines the special history and reality of grassroots government personnel and the requirements of national social governance. Based on this, this article attempts to pay attention to the phenomenon of the rules of favor in the grassroots government, first discusses the generation and impact of the rules of favor of the grassroots civil servants from a general level. This can also be better and provide inspiration for advancing the reform of the township administrative system. This article bridges the conflicts of sentiment, reason, and law in the social governance of grassroots government personnel, and explores the effective path of legal improvement and social governance.

## Materials and Methods

### Theoretical Basis

“Book of Rites” contains “what is human relationship? Joy, anger, sorrow, fear, love, evil, desire, and the seven are educated and capable.” As a term, early Confucianism regarded it as human instinctive emotions, referring to human natural and spontaneous feelings, which belonged to the organic part of human beings, and was a kind of mental state inherent in human beings. With the emphasis on ethics by Confucianism, the connotation of human relations has realized an important transformation from psychological knowledge to sociological knowledge. From the natural attributes of human relations, human relations can be arbitrary, intemperate, or unruly. Therefore, human feelings are transformed into a Chinese localized concept at the sociological cognitive level (Tan, 2019). In the pre-Qin period, the word of love was used to indicate the natural state of things or people’s minds, whether it refers to things, human sentiments or human nature (Fan et al., 2017). As Confucianism paid close attention to the issue of governance, human nature or human sentiment was used as a subsidiary issue of the rationality of governance discussing it has become a major topic that has always been of concern to people in Chinese academic history. “Human feelings” have three meanings: the first level of meaning refers to human emotions in a broad sense, such as personal emotions such as happiness, anger, sorrow, fear, love, evil, and desire, that is, personal emotions in interpersonal communication. The second level of meaning is the exchange of resources in reciprocity, referring to the “profit” contained in interpersonal communication. This kind of resource can be a materialized, tangible, actual existence, such as money, gifts and all other things that people need for food, clothing, housing, and transportation (He et al., 2019). It can also be non-materialized and intangible, manifested as an activity or process. The value of the two in the activities of interpersonal interaction is often difficult to objectively measure, the so-called “favorable debt” is the meaning of “unclear,” and the third meaning refers to the way of getting along with people in Chinese society, that is, the norms and norms that people should abide by in their interactions. The human affection ultimately falls on the moral “righteousness” in interpersonal communication.

Following the tradition of western administration, the conflict of administrative ethics is inevitable, and the conflict of power, interest, and role are typical manifestations of the conflict of administrative theory. The key to the instability of government order and the existence of conflicts lies in the fact that the ruling government establishes and maintains an unfair order. The frequent use of coercive force by the government will inevitably lead to the historical theory of violence cycle, which is the most uneconomic result. There are two aspects in dealing with moral conflict and realizing responsible moral behavior. The adoption of external control methods, that is, legal measures and organizational rules and regulations, requires internal control from the aspects of personal values and beliefs, that is, the conscious use of administrative staff. One’s own ethical autonomy resists the unethical and irresponsible behavior of the organization. Conflict theory is a theory that takes social conflict as the research object in modern and contemporary western sociology. Its formation indicates that conflict, as an inherent component of social structure and a basic form of social pattern, has received social attention (Pothier et al., 2019). People no longer avoid conflicts, but face them directly, and actively research methods to resolve conflicts. Contemporary conflict theories mainly include the dialectical conflict theory and the conflict functionalism. In the struggle for resource allocation, one party in the struggle wants to gain more from the limited power resources. Conflict, as an inevitable product of the development and changes of the relationship between man and society in the historical process, is manifested in the conflict of values. Its roots are the tendency of value subjects and the diversification of value subjects. In the process of public administration, the value conflict between value rationality based on fairness and justice and tool rationality based on efficiency is very common.

**FIGURE 1:**
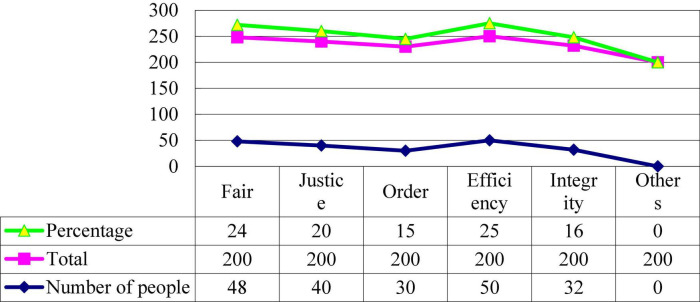
Investigation of factors involved in personal values.

**FIGURE 2:**
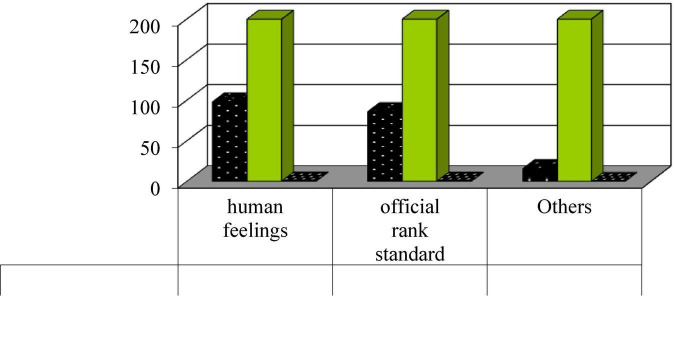
Cultural causes of administrative conflict.

Administrative agencies often pursue the realization of their own interests while pursuing the realization of public interests. Since this pursuit of interests has a close relationship with oneself, it is often more intense. Power conflicts such as “law enforcement fights,” “intersection of functions,” “multiple politics,” “power disputes,” “competition of interests and control, and disregard of interests” are very common, all of which reflect the power conflicts between government departments (Guzhavina, 2020). The universality and severity of administrative conflicts affect the overall efficiency of administration, shake the internal stability of the administrative system, and damage the government’s status and authority in people’s minds. In Figure 1, the efficiency accounts for 25%. Order accounts for 15%. Based on the interviews, everyone believes that the value system is not unique and will be adjusted according to actual changes (You, 2017). Fairness and justice in the implementation process have received widespread attention. The key is that it safeguards the public interest and embodies the “good purpose” of good governance. Its essence is to emphasize that grass-roots civil servants must perform their duties due diligence. The openness and “self-discipline” of the fair and just operating mechanism embodies the “means” of good governance and “good governance” itself embodies many values and principles pursued by mankind. In Figure 2, fairness accounts for 24% and justice accounts for 20%. Only with good governance as the goal or means can civil servants be right. Only by properly handling administrative ethical conflicts, putting democratic politics in the first place, and putting the interests of citizens in the first place, can we better serve the people. The implementation efficiency of grass-roots civil servants is generally believed to give top priority, and they said that while ensuring efficiency, they could try their best to ensure fairness and fairness. This shows to a certain extent that there is indeed a value conflict in daily work. More importantly, the current value system for grassroots civil servants has not really established.

### Building a Fuzzy Similarity Matrix

#### Data Collection

This paper selects 11 sites (_*x_i_*_) to conduct statistics on the administrative moral conflicts of grass-roots civil servants. The results are shown in Table 1.

**TABLE 1:** Feedback from the grassroots level collected in the past 10 years (copies).

No.	_*x*_1__	_*x*_2__	_*x*_3__	_*x*_4__	_*x*_5__	_*x*_6__	_*x*_7__	_*x*_8__	_*x*_9__	_*x*_10__	_*x*_11__
1	27	32	15	41	29	25	31	30	17	24	32
2	21	27	34	34	31	45	25	41	42	30	47
3	19	43	29	56	47	52	21	22	32	41	23
4	26	22	24	28	26	31	27	31	25	32	35
5	29	31	50	38	33	41	35	27	60	29	29
6	46	18	24	18	16	23	52	30	24	28	35
7	28	37	42	41	31	31	31	43	42	19	41
8	43	35	37	45	34	42	48	28	36	31	25
9	18	21	40	38	23	40	21	19	43	32	18
10	34	46	25	52	42	52	38	34	25	28	31

#### Building a Fuzzy Similarity Matrix

In this paper, the correlation coefficient method is used to construct a fuzzy similarity relationship matrix (f_*αβ*_)_11×11_, $f_{ij}=\frac{\sum_{k=1}^{n}\left|(x_{ik}-\bar{x_{i}})||(x_{jk}-\bar{x_{j}})\right|}{{\left[\sum_{k=1}^{n}{\left(x_{ik}-\bar{x_{i}}\right)}^{2}\cdot \sum_{k-1}^{n}{\left(x_{jk}-{\bar{x}}_{j}\right)}^{2}\right]}^{\frac{1}{2}}}$.

And ${}_{\bar{x_{i}}}=\frac{1}{10}\sum_{k=1}^{10}x_{ik}$, *i* = 1, 2, …, 11. $\bar{x_{j}}=\frac{1}{n}\sum_{k=1}^{n}x_{jk}$, *j* = 1, 2, …, 11.

Take *i* = 2, *j* = 1 and substitute it into the formula to get *f*_21_ = 0.839. Due to the huge amount of computation, the remaining values are calculated by C language programming, and the fuzzy similarity relationship matrix (*f*_αβ_)_11×11_ is obtained (Liu et al., 2021).

## Results

*T* is a symmetric matrix, so the lower triangular matrix is written as follows.


(1) $$T^{*}=\left[\begin{array}{ccccccccccc} 1.00 & & & & & & & & & & \\ 0.81 & 1 & & & & & & & & & \\ 0.67 & 0.67 & 1 & & & & & & & & \\ 0.81 & 0.96 & 0.67 & 1 & & & & & & & \\ 0.81 & 0.96 & 0.67 & 0.92 & 1 & & & & & & \\ 0.81 & 0.95 & 0.67 & 0.92 & 0.92 & 1 & & & & & \\ 0.94 & 0.81 & 0.67 & 0.81 & 0.81 & 0.81 & 1 & & & & \\ 0.79 & 0.79 & 0.67 & 0.79 & 0.79 & 0.79 & 0.79 & 1 & & & \\ 0.67 & 0.67 & 0.92 & 0.67 & 0.67 & 0.67 & 0.67 & 0.66 & 1 & & \\ 0.68 & 0.68 & 0.68 & 0.68 & 0.68 & 0.68 & 0.68 & 0.68 & 0.67 & 1 & \\ 0.79 & 0.79 & 0.67 & 0.79 & 0.79 & 0.79 & 0.79 & 0.68 & 0.67 & 0.68 & 1 \end{array}\right]$$

λ =0.96 is set, hence:


(2) $$T_{0.96}^{*}=\left[\begin{array}{ccccccccccc} 1 & & & & & & & & & & \\ & 1 & & 1 & 1 & & & & & & \\ & & 1 & & & & & & & & \\ & 1 & & 1 & & & & & & & \\ & 1 & & & 1 & & & & & & \\ & & & & & 1 & & & & & \\ & & & & & & 1 & & & & \\ & & & & & & & 1 & & & \\ & & & & & & & & 1 & & \\ & & & & & & & & & 1 & \\ & & & & & & & & & & 1 \end{array}\right]$$

The similarity is 1 under the threshold λ of *x*_2_, *x*_4_, *x*_5_ in the confidence level of 0.96 (Li et al., 2021). So they belong to the same category, so the observation stations can be divided into 9 categories at this time{*x*_2_, *x*_4_, *x*_5_ },{*x*_1_ }, {*x*_3_ }, {*x*_6_ },{*x*_7_ },{*x*_8_ }, {*x*_9_}, {*x*_10_ },{*x*_11_ }.

The confidence level is λ, and do the same analysis for different λ.

λ = = 0.95, it can be divided into eight categories, namely {*x*_2_, *x*_4_, *x*_5_, *x*_6_ },{*x*_1_ }, {*x*_3_ },{*x*_7_ },{*x*_8_ }, {*x*_9_ }, {*x*_10_ }, {*x*_11_ }.

λ = = 0.94, it can be divided into seven categories {*x*_2_, *x*_4_, *x*_5_, *x*_6_ },{*x*_1_, *x*_7_ }, {*x*_3_ },{*x*_8_ }, {*x*_9_ }, {*x*_10_ }, {*x*_11_ }.

_λ==  0.92_, it can be divided into six categories {*x*_2_, *x*_4_, *x*_5_, *x*_6_ },{*x*_1_, *x*_7_ }, {*x*_3_, *x*_9_ },{*x*_8_ }, {*x*_10_ }, {*x*_11_ }.

λ = = 0.79, it can be divided into five categories {*x*_2_, *x*_4_, *x*_5_, *x*_6_ },{*x*_1_, *x*_7_ }, {*x*_3_, *x*_9_ },{*x*_8_, *x*_11_ }, {*x*_10_ }.

## Discussion

### Factors Causing This Situation

The causes of the administrative ethics conflicts of grassroots civil servants under the social background of human relations have complex roots. The deep-rooted official standard thoughts, the insoluble human culture, the constantly changing system, and the influence of the economic and legal concepts of grassroots civil servants are the causes of human relations. The main reason for the administrative ethics conflicts of civil servants at the social background.

#### The Influence of Human Relations Culture and “Official Standard” Thought

China’s “official standard” ideology is deeply ingrained, and most of the civil servants at the grass-roots level are located in urban areas with more traditional culture. In these places, the ideology of “finding relationships and emphasizing human relations” is deep-rooted, and culture has a more profound impact on grass-roots civil servants. Due to the influence of the “official standard” thinking, being an official has become the goal of people’s pursuit of struggle. A large number of talents flock to government departments, which is likely to cause an unreasonable allocation of human resources in the whole society. Under the dominance of the “official standard” thinking, there will often be a phenomenon that “rule of man is greater than system.” “Human relationship,” administrative power and the will of chief executives appear in decision-making from time to time, restricting those capable talents to a certain extent. Outside of the core departments, this situation not only weakens the efficiency and service level of government departments, but also reduces the enthusiasm of some practical talents, is not conducive to healthy competition within the department, and will cause serious internal consumption of human resources. Under the influence of the “official standard” consciousness, some unit leaders are above the top instead of the bottom, and they are easily separated from the masses. They are keen to keep their positions and follow the old fashioned and not enterprising. They are not good at making innovations based on the changed market environment. With limited administrative resources, many talents are not diligent in studying professional skills, but painstakingly studying “the way to be an official and the key to politics,” which is resulting in vicious competition among unit talents and serious internal friction; some are devoted to professional skills. The talents of teaching, scientific research or production and management are often restrained by the administrative will of the official standard. These have curbed the excavation and exertion of human resource creativity.

Among those surveyed, most people think that social factors have led to the unsmooth administrative work system according to law. This part of the factors accounted for 49%. The competent authority for administrative work according to law in our country is the legal institution of the government at all levels, which bears the important responsibility of formulating and organizing the implementation of administrative work according to law. Some public servants have serious formalism and are accustomed to traditional ways of thinking and work. They despise legal means and emphasize individual handling, and system management emphasizes coordination. The typical phenomenon of officialdom culture is serious, and the influence of officialdom culture on administrative execution accounts for 43%. Other factors accounted for 8%. It can be seen that in the context of human relations and society, most people believe that the Chinese human culture and official status are important cultural factors that affect administrative ethics.

#### Human Relations Transformed Into Economic Benefits and Reciprocity

In essence, the law of human relations embodies a kind of “repaying” thinking. As we all know, human society has the behavioral logic of “repaying” since its inception, and has become a basic code of conduct recognized by almost any culture. In particular, the law of human relations in Chinese culture is a derivative of the “regulation of retribution.” The interpersonal relationship under the law of human relations is also often fragile, because the prerequisite for maintaining the human relations under the law of human relations is that both parties must pay attention to “investing in peaches and repaying them with plums” and “reciprocity.” When one party asks for help, the other party will consider making a rational expectation of the return that the other party may make. It based on the expectation that people “behave favorably” to others. In the interpersonal interactions of Chinese society, human feelings can be transformed into economic benefits and reciprocity, the social status and prestige that individuals have achieved in society. It can be seen that human relations essentially exist as a way of resource diffusion. The personal dignity of a grassroots official comes from his correct behavior and the social praise he has received. In a market economy, human affection is money. Wherever there is interest, human affection may play a role. The grass-roots civil servants hold social public resources. These social resources can be used as a kind of value-added capital for benefit exchange, which become grass-roots civil servants. Turning favors into economic benefits and reciprocal bargaining chips, the benefits of these bargaining chips are usually higher.

#### Imperfect Systems and Mechanisms Leave Room for Human Relations Operations

In the process of economic transformation, the grassroots government holds a lot of public power. According to the logic of bureaucracy, the exercise of public power should be done according to the rules and without discrimination. However, in addition to the public responsibilities entrusted by public office, government officials who specifically grasp and exercise public power also have human responsibilities stipulated by the human society. The former requires “selflessness” and the latter requires “concealment of relatives.” In the case that the internal responsibility restraint mechanism of the public power system is not perfect, the conflict between these two responsibilities is often easy to fall to the victory of the latter, because the “inhumane” people are often rejected by the human society, and thus the power is rejected. The exerciser causes huge social pressure. In this kind of social atmosphere, the corruption of public power seldom manifests itself as a naked “transaction of power and money,” and it is more about appealing to favors, establishing and consolidating personal relationships through long-term “reciprocity.” Even when public power is used normally, it often needs to be lubricated through human relations. In the current system, government departments have overlapping functions and multiple politicians. This is an important reason that causes various departments to buckle and buckle each other, leaving room for human relations. Many things should have handled strictly and quickly with laws and regulations. It will become individual leaders as “human feelings” and handle them flexibly, so that the public interest cannot have effectively maintained, and it is easy to cause the psychological confusion of grassroots civil servants, whether it is the priority to obey the orders of department leaders or the orders of other department leaders. In current assessment system, the assessment of grassroots civil servants emphasizes political and efficiency considerations, requiring grassroots civil servants to implement policies and resolutely obey orders from their superiors (Wu et al., 2022a,b). Instead of fully considering the quantitative assessment of safeguarding public interests, although this design can ensure the implementation of national policies, but it also gradually weakens the concept of fairness and justice for basic-level civil servants, which is not clear enough, and is likely to cause administrative ethical conflicts. Supervision is a kind of heteronomy and an external requirement for the performance of basic-level civil servants. In our country’s administrative accountability, there is a serious rule of man. Most of the accountability is based on organizational intentions or leaders’ instructions. There is a lack of necessary legal protection. It is precisely because of the lack of effective power supervision and restriction that human sentiment is detrimental to public administration. The impact is difficult to eliminate. With the continuous advancement of China’s legalization process, establishing an effective supervision mechanism, China’s administrative agencies have made active and useful explorations.

#### Lack of Autonomy in Public Administration Ethics of Basic-Level Civil Servants

It is undeniable that when basic-level civil servants exercise public power, they will inevitably have affected by culture, economy, and system, but they will also have affected largely by the basic-level civil servants themselves. Human nature has a dual nature, that is, there are both rational and irrational aspects. As one aspect of human nature, human reason is time-sensitive and does not always remain rational; as another aspect of human nature, human irrationality is blind, Impulsive characteristics, people’s thoughts and behaviors cannot be purely rational or purely irrational, but are the result of the interaction between the two. In various administrative activities, basic-level civil servants are not unconscious actors. They need to make value judgments and ethical choices based on policies, regulations and higher-level requirements, as well as their own experience and experience, and bear corresponding moral responsibilities. This subjective judgment and ethical Choice is the autonomy of administrative ethics. The ethical autonomy of basic-level civil servants depends on three aspects, namely, the understanding of laws and administrative ethics, the perception of human society, and the strength of their own willpower.

Our country is actively advancing the construction of the rule of law, and its fundamental requirement is that the administrative body governs the country in accordance with the law. However, some grassroots civil servants have weakened their legal consciousness, and the hedonism and money-oriented unhealthy trends have spread. “The principle of dealing with things,” in a human society, like to play “side ball,” which results in administrative ethical conflicts. Individual basic-level civil servants have misunderstandings on administrative ethics, and their sense of responsibility has weakened. “Be responsible,” ignore the interests of the masses, place too much emphasis on the “official standard” and forget that “all power comes from the people.” As the “agents” of the masses, grassroots civil servants must express public will instead of mixing private will and seeking personal gain. If there are no errors and deviations in administrative ethics, there will be no administrative ethical conflicts.

**FIGURE 3:**
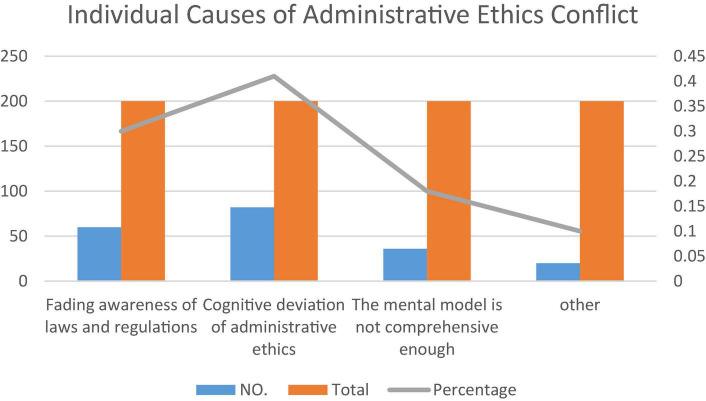
Individual causes of administrative ethics conflict.

Individual reasons in administrative conflicts will be brought to the entire problem management formulation, and individual reasons breed the tendency of individual interests, so that collective rationality suffers, and individual behavior disrupts the fair and reasonable development of administrative management problem programs. As shown in Figure 3, 40% of people believed that the individual cause of the conflict is the cognitive deviation of administrative ethics. Secondly, many grassroots cadres fail to realize the legal nature and consequences of their actions when improper use of power has caused serious consequences. This is a concentrated manifestation of the lack of legal knowledge; the weakened awareness of laws and regulations accounts for 32% (You, 2017). Looking at the current grassroots leading cadres, they often act in a half-knowledge and self-righteous manner, they do not follow the law, do not abide by clear rules and regulations, and like to act in the usual way. Nineteen percent of people think that the mental model is not comprehensive enough. Others accounted for 9%. It can be seen that, for basic-level civil servants, the lack of their own legal awareness and the cognitive deviation of administrative ethics are the biggest obstacles to their compliance with administrative ethics and avoid falling into administrative ethical conflicts.

### Governance Countermeasures

Resolving the administrative ethics conflicts of civil servants at the grassroots level is a complex project that involves society, administrative organizations, and civil servants at the grassroots level, as well as cultural and institutional issues. There are two ways of solving administrative ethical conflicts. One way is to transform the external environment, reduce the space for human affection to interfere with public administrative behavior, and create a good social environment; the other way is through internal system reform and administrative education to promote the basic-level civil servants to independently resolve administrative ethical conflicts with their perfect personality.

#### Governance Adjustments to Avoid Administrative Ethics Conflicts Among Grassroots Civil Servants

The so-called “free-riding” and “back door” of the human relationship in the human relationship society conflict with the equality before the law advocated by the contract model, and the implementation of laws and policies cannot be arbitrarily flexible. Probably, the legacy of a country with great human sentiment in China can have suppressed only by the improvement of the real market system and the baptism of political system reform. Our country is in a period of social transformation, and the human society has become a shackle. To get rid of this kind of human feelings, the key is to transform the traditional human society, build a contract-based society, and let the “contract concept” take root in the hearts of the people. Grassroots civil servants should increase the intensity of legal aid in law enforcement, and increase the popularization and specific interpretation of existing laws and regulations through various forms and methods, so that ordinary people have the law in their hearts, respect the law in their hearts, and can know the law. The second is to improve the education level of basic-level civil servants, then improve the political, legal, and governance literacy of minority cadres, and invite basic-level civil servants with higher education and good Mandarin to study and interpret laws and regulations, to integrate existing laws in the daily life and habits of basic-level civil servants. The third is to update and interpret in time in light of the development of the times and the needs of society. No matter in any era, the law lags behind the development of society, so the laws and regulations must not be constant. They need to update and supplement in a timely manner. It is necessary to make appropriate interpretations and even adaptations when dealing with specific problems and specific areas. The fourth is to strengthen law enforcement, ensure the quality of law enforcement personnel, improve the treatment of law enforcement staff, and increase their enthusiasm for work. Based on the existing establishment, appropriately develop non-governmental forces to assist law enforcement, and change unilateral government actions into society and individuals. Multiple co-governance attracts more people to participate in the social governance of civil servants at the grassroots level.

With the help of rural sage governance, we must guide and encourage modern rural sages to participate in social governance in the current context, to realize the organic governance of rural sages and the rule of law in society. Since the religious power of grassroots civil servants is entangled, the influence of religion on the local people should not be underestimated (Yan et al., 2020). Religious and religious activities should have properly guided and regulated to make them more in line with the requirements and direction of the construction of a harmonious society, and rely on the powerful influence of religion and the ability to control resources. In the process of rule of law, the first is to strengthen the legislative construction of grassroots civil servants, and institutionalize the rule of law in terms of legal provisions. The second is to implement the implementation of national and local laws and regulations on grassroots civil servants. Only the law that is enforced is the law that really works, otherwise it is just a dead letter. The third is that legal principles are nothing more than human sentiments. Social governance requires the coordination and cooperation of emotions, reasons, and laws. Based on understanding and respecting the cultural habits and national traditions of basic-level civil servants, the laws and regulations are detailed in a way that is more acceptable to local people. The fourth is to keep pace with the times, and constantly update and innovate the content, form, method, and approach of the legalization of grassroots civil servants. Therefore, to transform the human society, it is necessary to strengthen the ability-based awareness, so that the public can focus on work and career, and give full play to their abilities. As far as the agency is concerned, it can strengthen its administrative culture construction, implement the “both ability and political integrity” criteria for selection and employment, and improve the performance evaluation mechanism. The traditional misconception that only relying on human relations can be promoted at the grassroots level, so that it can help grassroots civil servants get rid of the shackles of blood ethics and hierarchical status, and get out of the vicious circle of power corruption caused by the role of human relations.

#### Deepen the Reform of Government Governance and Establish an Administrative Ethical Incentive Mechanism for Grassroots Civil Servants

Just as there is no uniform and unchanging model in modern society, the basic administrative system has diversity and complexity. The reason why renqing can interfere with the administration of grassroots civil servants in accordance with the law is mainly manifested in preventing the normal implementation of the system. In this regard, it is necessary to ensure that renqing operates under the premise of complying with laws and systems to avoid adverse effects caused by human factors. Improvement of laws and regulations is fundamentally a code of conduct for regulating and restricting the behavior of groups or individuals. The idea of official status in China has existed for a long time, largely because the system is not scientific and reasonable. Only when rewards and punishments are combined, the role of laws and regulations can be maximized, so that people who abide by ethics and ethics can be protected and protected. Rewards will deter those who want to use power for personal gain, and those who use power for personal gain will have severely punished. The survey shows that more than 80% of officials, especially grassroots officials, have greater psychological pressure, mental fatigue, and psychological imbalance. These pressures not only endanger the physical and mental health of leading cadres, but also affect their leadership performance and family harmony (Hossain, 2020). Part of these pressures comes from within the organizational system, that is, when the values, thinking styles, abilities, motivations, personality and behavior styles of the leaders themselves are incompatible with the organizational structure, processes, systems, and leadership styles, the higher the degree of mismatch between the two. The greater the work pressure. Especially when the ethical behavior of leading cadres is not supported by the organization, individuals will be “incompetent,” triggering a series of physical, psychological and behavioral changes that affect their work performance.

For grassroots leading cadres, how to maintain a sustainable ethical form through the recognition and support of the organization requires a built-in dynamic guarantee mechanism, which is an incentive mechanism. At present, there is a dislocation in the incentive mechanism for the ethical behavior of grassroots leading cadres. For example, those leading cadres who show good moral behavior may have seriously damaged in life, career, reputation, and personal economic income. Therefore, the establishment of a “profit promotion” mechanism enables organizations or individuals that abide by ethical behaviors to gain higher benefits than they lose due to ethical behaviors, that is, to reduce the net benefits that leading cadres bring to themselves or the organization from their misconduct. At present, the most important incentive mechanism for grassroots leading cadres is the fair and orderly selection system, because many of the behaviors of grassroots cadres occur in the promotion process. Under the current selection system, people with official ethics may not necessarily benefit from it. Therefore, the establishment of a cadre selection system with “consistent morality and happiness” and its corresponding operating mechanism doomed civil servants to weigh the pros and cons. Only honest and ethical behavior is the wisest choice. Then, it is not very important for civil servants to keep the moral bottom line. If this kind of institutional arrangement is stable, public servants will form a stable behavior pattern in this stable behavior selection process. Over time, this stable behavior selection will become a habitual behavior, that is, consciously consider the legitimate interests of the individual in the context of the requirements of the public interest of the society. Therefore, building a good environment in which people who practice moral behavior do not suffer at a macro level, and ensuring the integrity of virtue is the moral bottom line for the selection and promotion of cadres (Nakayama et al., 2020). When the benefit of a leading cadre’s integrity is much greater than its cost or risk, he has a motive of integrity. When the benefit of such integrity is large enough, he may become a professional government official and produce a radiation effect that promotes integrity. Therefore, an organizational atmosphere will have formed.

Improving welfare benefits is an objective requirement for resolving the administrative ethics conflicts of grassroots civil servants. From the perspective of the external environment, administrative grassroots civil servants are all in the county-level and below-county administrative areas, and the wage level has linked to the local finances, resulting in low wages and low positions. In recent years, the state has introduced policy inclination in salary level adjustment and vehicle reform, which has improved the benefits of basic-level civil servants to a certain extent. However, compared with enterprises, the salary level of most basic-level civil servants is not high. The evaluation of social status is high, and it is easy to pursue material enjoyment. If the actual wage level cannot satisfy their desires, there will often be the phenomenon of using power to trade power and money. Therefore, it is necessary to establish a reasonable welfare mechanism for civil servants and appropriately increase the welfare benefits of grassroots civil servants. They must not stay at the level of spiritual benefits, but must enter the level of economic benefits, so that grassroots civil servants do not have to run for their livelihoods and suppress their motivation to seek gray income. Most basic-level civil servants have professional ideals for position promotion. If there is no reasonable promotion channel, their position demands cannot have met, which will easily lead to the use of human relationships to win promotion capital (Chen et al., 2020). Therefore, the promotion mechanism should have improved to meet their promotion needs.

#### Strengthen the Administrative Ethics Education of Basic-Level Civil Servants, and Provide Ideological Guarantee for the Correct Use of Power by Basic-Level Civil Servants

Basic-level civil servants have dual role identities and natural weaknesses of human nature. To enable them to exercise public power when facing administrative ethical conflicts, it is far from enough to rely on external control. Just imagine that a good system is in the political economy. It is important. Administrative ethics education is an important way to cultivate the ethical character of administrative subjects. A perfect administrative ethics education system is the institutional guarantee of administrative ethics and administrative ethics education. The establishment of a basic-level civil servants’ administrative ethics education system is a macroscopic and universal problem, but it must be close to the local basic-level civil servants in many links. The public administration department should also design relevant administrative ethics training for newly recruited and promoted civil servants. In terms of training, private companies have done a very good job. Although there is a big difference between public units and private units, they can also learn from each other in many places. Of course, public administration units learn from private units. Staff training should be divided into two parts: induction training and promotion training.

The public sector should join the corresponding administrative ethics training when public employees enter their jobs. The content of the training can be divided into two parts: theory and practice. In this part of theory, colleges and universities can hire teachers with relatively high standards in administrative ethics research and teaching to explain to the recruits. In practice, the relevant leaders and trainers within the department can be responsible for sharing experience with new civil servants from actual work, such as how to think and act as a grassroots civil servant when facing role conflicts. Before being promoted, the professional ethics level of the personnel should be tested and the administrative ethics training should be further strengthened for qualified personnel. Of course, administrative ethics training is not limited to the time of entry and promotion, and the assessment of compliance with administrative ethics should be included in the annual year-end assessment. All of the above are the routine training of administrative ethics. Local grassroots departments can also explore more flexible and diverse training methods according to their own actual conditions. For example, the “cadre recall” system currently implemented in many places is a manifestation of the promotion of a comprehensive extension of the strict governance of the party to the grassroots. It is also a training method in administrative ethics. The law can solve illegal and criminal acts such as corruption, and the “cadre recall” system mainly focuses on the ideological education of cadres and solves the problem of “inaction.” In short, in the context of my country’s complex reforms, while the external control of system anti-corruption and the rule of law and anti-corruption, attention should be paid to the ideological education of basic-level civil servants, and the establishment of a training system for basic-level civil servants’ administrative ethics education can improve civil servants’ recognition of their own professions. Knowledge, establish the correct professional outlook and values, eliminate professional burnout, thereby optimize their professional behavior, promote the realization of public sector interests and the cultivation of public value.

Once the system is established, to a certain extent, people who want to “trust favors and relationship” cannot achieve the established goals, and it can also eliminate the difficulty of ethical choices of grassroots civil servants (Zhang et al., 2020). However, the role of the system is limited. Soundness, because of the existence of interests, there will be people who seek favoritism and seek personal gain. Therefore, it is very necessary to improve the awareness of administrative ethics conflicts. When dealing with administrative ethical conflicts, laws and regulations are one of the first choices. However, laws and regulations cannot cover everything and cover all situations of public administration. When basic-level civil servants make ethical choices, personal morality is very important. To strengthen the moral cultivation of grassroots civil servants, on the one hand, it is necessary to help them establish a correct concept of power, status and human feelings, strengthen the awareness of right and wrong and rules, keep in mind the purpose of serving the people, and reduce the constraints of human factors. On the other hand, reshape the administrative conscience of civil servants at the basic level (Agyei-Mensah, 2017). Administrative conscience is an internal psychological reaction mechanism for civil servants to make self-ethical evaluation of their professional behavior. In terms of individual needs, the behaviors of basic-level civil servants come from his desires and goals. If the moral and non-moral motives are consistent, he will abide by administrative ethics, but if there is a conflict, the basic-level civil servants will often choose to satisfy with great desires and goals. If the grassroots civil servants have a strong willingness to administrative conscience, they can follow the administrative conscience, correct their thinking, give up personal interests, and safeguard public interests. Therefore, it is also very important to cultivate the administrative conscience of basic-level civil servants.

**FIGURE 4:**
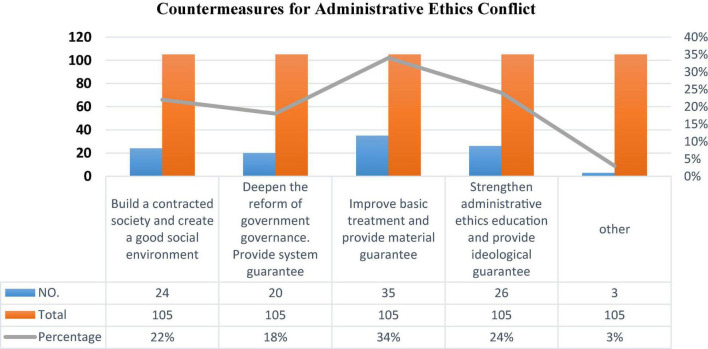
Countermeasures for administrative ethics conflict.

Basic-level civil servants are mostly faced with the people at the grass-roots level. They are aware of the most real needs of the people, and it is relatively hard to go to the mountains and the countryside. Not only that, the promotion of civil servants at the grassroots level is relatively slow. Without a higher level, the salary level cannot be improved. Even after working for several decades, it is difficult to improve the salary. If things go on like this, it will be difficult to retain young and promising talents. After all, civil servants are also human and need to support their families. In the survey sample (Figure 4), 34% need to improve the basic treatment. After the salary of basic-level civil servants is increased, the management method becomes more flexible and scientific. Especially the flow of professional and technical talents, they have professional knowledge and rich practical experience behind them. The influx of such talents into all civil servants will improve the overall professional and technical level of our civil servants and better serve the economic and social development. The transformation of professional and technical personnel’s job roles requires strengthening administrative ethics education, which accounts for 24%. The performance of the organization’s contract has an impact on the evaluation of the effectiveness of incentive measures by basic-level civil servants. In an environment where the contract is not well-fulfilled, material guarantees have a stronger incentive for basic-level civil servants, and basic-level civil servants tend to ignore emotional incentives and pay more attention. Among the investigators, contract society and deepening government governance reform accounted for 22 and 18%. Most grassroots civil servants believe that improving grassroots treatment and strengthening administrative ethics education are important countermeasures to resolve contradictions.

## Conclusion

The social phenomenon of human relations in the grassroots government is a manifestation of the dual identities of the “locality” and “bureaucratic” of the township cadres in their behavior. It is also the daily routine formed by the township cadres based on the characteristics of the local politics and their own political needs under the framework of the administrative ethics. The code of conduct is one of the main reasons for the alienation of the behavior of township cadres. Therefore, in the process of advancing the reform of the township administrative management system, it is necessary to pay attention to the social phenomenon of human relations in the grassroots government, and to deal with it from the following aspects. First, in the concept of government management, we should rationally recognize the objective facts of the existence of human relations and society, and treat township cadres with a more flexible and pragmatic attitude. The environment gives it enough room for discretion. Village and township officials sometimes make use of human relations and society to improve administrative efficiency. Secondly, in the construction of social culture, we should actively promote the transformation of rural culture. The human society is the social and cultural root of the grassroots government. Therefore, we promote the transformation of the local culture from closed, perceptual, and humane to open, rational, and rule-oriented, and provide a favorable external environment for the reform of the grassroots government. Thirdly, in the personnel management system, on the one hand, we must continue to increase the education and training of township cadres, focusing on changing concepts, improving quality, and adjusting roles, and combining with the recent opportunities of mass line education, actively establish service-oriented grassroots cadres. On the other hand, we will continue to adhere to and improve the examination system of grassroots civil servants, attract more management talents with modern government management concepts for the grassroots government, and reduce the informal rules phenomenon in the grassroots government by optimizing the talent structure of the grassroots government.

## Recommendations

As the direct implementers of government public policies, grassroots civil servants play a pioneering role in promoting economic development and social stability. Grassroots civil servants will inevitably encounter various administrative ethics conflicts. This paper pays close attention to the administrative ethics conflicts encountered in the process of policy implementation of grassroots civil servants by fuzzy cluster analysis, and puts forward a set of reasonable and effective administrative ethics system to help grassroots civil servants correctly deal with various contradictions. This article based on authority conflicts, role conflicts, conflicts of interest, value conflicts, power reproduction, etc. Based on this, it puts forward the countermeasures for the administrative ethics conflicts of grassroots civil servants under the social background of human relations, namely, to build a contract-based society, deepen the reform of government governance, improve the benefits of grassroots civil servants, and strengthen administrative ethics education, etc. Through the discussion of this article, it provides some theoretical support for the administrative ethical conflict and governance of civil servants in our country under the background of human relations and society, and provides some useful reference for the basic-level civil servants to deal with and solve the administrative ethical conflict.

## Data Availability Statement

The original contributions presented in the study are included in the article/supplementary material, further inquiries can be directed to the corresponding author.

## Ethics Statement

The studies involving human participants were reviewed and approved by the Mudanjiang Medical University. The patients/participants provided their written informed consent to participate in this study.

## Author Contributions

YY, TL, and FY contributed to conception and design of the study. TL organized the database. FY performed the statistical analysis. YY wrote the first draft of the manuscript. TL and FY wrote the sections of the manuscript. All authors contributed to manuscript revision, read, and approved the submitted version.

## Conflict of Interest

The authors declare that the research was conducted in the absence of any commercial or financial relationships that could be construed as a potential conflict of interest.

## Publisher’s Note

All claims expressed in this article are solely those of the authors and do not necessarily represent those of their affiliated organizations, or those of the publisher, the editors and the reviewers. Any product that may be evaluated in this article, or claim that may be made by its manufacturer, is not guaranteed or endorsed by the publisher.
